# First Directly Sequenced Genome of Hepatitis E Virus from the Serum of a Patient from the United Kingdom

**DOI:** 10.1128/genomeA.00931-16

**Published:** 2016-09-15

**Authors:** Sarah L. Caddy, Ian Goodfellow, Hamid Jalal

**Affiliations:** a Department of Pathology, Division of Virology, University of Cambridge, Cambridge, United Kingdom; b Public Health England, Cambridge, United Kingdom

## Abstract

Hepatitis E virus (HEV) genotype 3 is endemic in the United Kingdom but the complete sequence of HEV, generated directly from a clinical sample, is lacking. We report a near full-length genome sequence of genotype 3 HEV from the serum of a patient with acute hepatitis.

## GENOME ANNOUNCEMENT

Hepatitis E virus (HEV) is the most common cause of acute viral hepatitis in the United Kingdom. The frequency of detection of acute HEV infection was reported to be 31-, 9-, and 12-times higher than that of hepatitis A, B, and C, respectively (1). About half of acute hepatitis E cases in England and Wales were reported to be autochthonous infections (2–5). At present, there is only one complete genome sequence of human HEV in GenBank from the United Kingdom, cell-adapted Kernow-C1 strain (6). A near full-length genome sequence of HEV was obtained from an anonymized residual serum sample from a patient with acute hepatitis from England, strain SLC-4.

RNA was extracted from 200-µl serum using the GenElute Mammalian Total RNA miniprep kit (Sigma Aldrich), and then treated with DNase (Roche). cDNA was synthesized using SuperScript III reverse transcriptase (Thermo Fisher). The viral genome was amplified in four segments using a nested PCR approach with a touchdown cycling protocol as previously described (7). Sequences at the extremity of the viral genome were determined by using 5′ and 3′ rapid amplification of cDNA ends (RACE) (Life Technologies). Sanger sequencing was performed using 16 primers covering the HEV genome (7). Overlapping sequences were assembled by the Vector NTI Express Designer software (Thermo Fisher Scientific).

A sequence of 7,229 bp in length with G+C content of 55% was generated from SLC-4. While 5′ RACE provided some sequences of the 5′ end of the HEV genome, due to the limited volume of clinical sample, we were unable to determine the 25 nucleotides of the 5′ untranslated region (UTR) and the first 18 nucleotides of open reading frame 1 (ORF-1). The genomic organization of SLC-4 was similar to those of other genotype 3 HEVs: ORF-1 (up to nucleotide [nt] 5,094), ORF-2 (nt 5,129 to 7,114), ORF-3 (nt 5,091 to 5,459), and 3′ UTR (nt 7,115 to 7,229). The nucleotide similarity of 90% and 86% was found between SLC-4 and sequences recovered from wild boars from Germany (FJ705359) and Mongolia (AB290312), respectively. The closest HEV genome (89%) recovered from a human was from a chronically infected kidney transplant recipient in southeastern France (KJ701409). Eighty-four percent homology was found between SLC-4 and Kernow-C1.

About 15% of pig meat consumed in the United Kingdom is imported (8), giving rise to the possibility of an enormous genomic diversity among locally prevalent strains. Homology between SLC-4 and other human and pig strains shows the extent of their genetic relatedness. However, due to the lack of genomic data, we are unable to examine the genetic relatedness of SLC-4 and strains prevalent in the United Kingdom. British epidemiological studies, quoted above, are based on sequencing data from a 304-nt segment of ORF-2 (2, 3), providing only a limited amount of information about the magnitude and nature of genomic diversity among HEV strains circulating in the United Kingdom. A comprehensive genetic analysis of HEV strains from the United Kingdom human and animal reservoirs is critical to gain a better knowledge of the diversity and epidemiology of this virus.

### Accession number(s).

The sequence is available in GenBank under accession no. KX462160.
